# Comparison of the outcomes of *in vitro* fertilization and embryo transfer among ethnic Chinese Yi and Han women: a multicenter retrospective cohort study

**DOI:** 10.7717/peerj.21145

**Published:** 2026-04-17

**Authors:** Jiexiu Chen, Xin Tan, Liangbo Yang, Xingyu Lv, Jiajing Wei, Shiqi Chen, Furui Chen, Wei Zhang, Jiuzhi Zeng, Shaomi Zhu, Jesse Li-Ling, Yan Gong

**Affiliations:** 1 Department of Clinical Pharmacy, Sichuan Provincial Women’s and Children’s Hospital, The Affiliated Women’s and Children’s Hospital of Chengdu Medical College, Chengdu, China; 2 Reproductive & Women-Children Hospital, Chengdu University of Traditional Chinese Medicine, Chengdu, China; 3 Sichuan Jinxin Xinan Women and Children’s Hospital, Chengdu, China; 4 Reproductive Medicine Center, Sichuan Provincial Women’s and Children’s Hospital, The Affiliated Women’s and Children’s Hospital of Chengdu Medical College, Chengdu, China; 5 Department of Biostatistics, School of Public Health, Fudan University, Shanghai, China; 6 Reproducive Medicine Center, Chengdu Fifth People’s Hospital, The Second Clinical Medical College, Affiliated Fifth People’s Hospital of Chengdu University of Traditional Chiese Medicine, Chengdu, China; 7 Department of Medical Genetics, West China Second University Hospital, Sichuan University, Chengdu, China

**Keywords:** *In vitro* fertilization, Embryo transfer, Race, Ethnicity, Live birth

## Abstract

**Background:**

Live birth rate (LBR) has been associated with ethnicity and race in Europe and the United States. However, the impact of ethnicity on LBR among multiethnic Chinese infertile women has remained unexplored. This study aims to investigate whether LBR of *in vitro* fertilization and embryo transfer (IVF-ET) is different between ethnic Yi and Han Chinese women.

**Methods:**

A total of 22,450 Han and 863 Yi women aged 20~45 years undergoing the first cycle of IVF-ET at three tertiary hospitals were retrospectively enrolled in this study. The protocols of ovarian stimulation were gonadotropin-releasing hormone (GnRH) agonist and GnRH antagonist, and the primary outcome was LBR. To minimize the influence of confounding factors, 1:1 propensity score matching (PSM) analysis was carried out before the outcomes of IVF-ET were analyzed. This clinical trial was registered with the Chinese Clinical Trial Registry (Registration number: ChiCTR2300070269).

**Results:**

After PSM, the rates of implantation, clinical and ongoing pregnancy, and LBR of Yi women (*n* = 853) were significantly lower than those of Han women (*n* = 853) (*P* < 0.05). Subgroup analyses further indicated that the impact of ethnicity on LBR was most significant in women aged 20~35 years (adjusted odds ratio (OR) = 0.77, 95% confidence interval (CI) = [0.62-0.97]) and in women with normal body mass index (adjusted OR = 0.76, 95% CI [0.58-0.98]). Multiple regression analysis revealed that age was associated with LBR in both ethnicities, while body weight may also affect LBR in Han women (*P* < 0.05).

**Conclusions:**

The outcomes of IVF-ET were worse in Yi women compared to Han women. Ethnicity and age are associated with LBR. Maintaining a normal weight may be beneficial for LBR, especially for ethnic Han Chinese women.

## Introduction

Infertility is a global and multiethnic problem, and people of color are less likely to achieve pregnancy after autologous *in vitro* fertilization (IVF) compared to White women (Boivin et al., 2007; Beroukhim & Seifer, 2023). Sharara & McClamrock (2000) noted that Black women had a significantly lower implantation rate (IR; 9.8% *vs*. 23.4%) and clinical pregnancy rate (CPR; 19.2% *vs*. 42.2%) compared to White women during *in vitro* fertilization and embryo transfer (IVF-ET). Langen et al. (2010) reported that Asian women undergoing blastocyst transfer had a significantly lower live birth rate (LBR) compared to White women even after multiple analysis (adjusted odds ratio (OR) = 0.48, 95% confidence interval (CI) = 0.24–0.96, *P* = 0.04). A recent review also identified ethnicity as a predictor for live birth after IVF (Shingshetty et al., 2024), while two other studies excluded the association between LBR and ethnicity (including White, African American, Hispanic, and Asian women; Lee et al., 2023; Bendikson et al., 2005).

There are 56 ethnic groups in China, each with different living customs, sanitary conditions, living environments, and eating habits. The outcomes of IVF-ET may be different among ethnic groups, though only a few retrospective studies on this topic have been conducted. Zhang et al. (2023) reported that CPR and LBR were similar between ethnic Mongolian (*n* = 622) and Han Chinese women (*n* = 4,586) undergoing fresh and frozen embryo transfer in the Central and Western parts of Inner Mongolia. Huang (2023) found that CPR was similar between ethnic Zhuang (*n* = 349) and Han (*n* = 481) women with polycystic ovarian syndrome undergoing fresh embryo transfer. Lin & Zhang (2018) reported that CPR and LBR were similar between ethnic Han (*n* = 1,089) and ethnic minority women (*n* = 51) from a single reproductive center. Of note, these studies were limited by their sample size, inadequate adjustment for confounding factors, selection bias, and extensive missing data.

According to the latest China population census, among the 34 provincial-level administrative regions in the country, Sichuan ranks the fifth largest and holds 5.93% of the national population (Office of the Seventh National Census Leading Group of The State Council, 2022). It is also a diverse province with 53 ethnic groups, among which ethnic Hans make up 93.2% of the total population, followed by ethnic Yis at 3.9%. A retrospective multi-center study was conducted to compare the outcomes of IVF-ET between ethnic Han and Yi women in Sichuan Province.

## Materials and Methods

### Study population

Ethnic Yi and Han Chinese women (determined by identity card) aged 20~45 and undergoing IVF-ET between January 2014 and December 2022 were retrospectively enrolled from three tertiary hospitals (Sichuan Provincial Women’s and Children’s Hospital, Reproductive & Women-Children’s Hospital of Chengdu University of Traditional Chinese Medicine, and Sichuan Jinxin Xinan Women and Children’s Hospital). Only couples of the same ethnicity were included. The three hospitals are all located in Chengdu, Sichuan province. All patients had undergone the first cycle of IVF or intracytoplasmic sperm injection (ICSI) with non-donor oocytes and sperm. The ovarian stimulation protocols were gonadotropin-releasing hormone (GnRH) agonist or GnRH antagonist. Patients were excluded for any of the following conditions: cancelled oocyte retrieval or fresh embryo transfer, preimplantation genetic testing, presence of uterine fibroids, frozen oocyte, ICSI with testicular sperm aspiration, three embryos transferred, and incomplete information of IVF-ET. This study followed the guidelines of the Declaration of Helsinki for Medical Research involving Human Subjects (2013 revision) and was conducted in accordance with the Medical Ethics Committee of Sichuan Provincial Women’s and Children’s Hospital (No. 20230216). Informed consent was waived given the retrospective nature of the study.

All information was derived from the patients’ electronic medical records. The etiology was defined as the main factor that induced infertility, though mixed factors may exist. Height and weight were measured, and body mass index (BMI) was calculated as weight divided by height squared (kg/m^2^). Serum levels of estradiol (E_2_) and progesterone (P) were measured using an electrochemiluminescence immunoassay platform (Roche Diagnostics GmbH, Mannheim, Germany). Intra- and inter-assay coefficients of the above variation were <5% and 10%, respectively.

### Ovarian stimulation

For the GnRH antagonist protocol, 100~300 IU/day of recombinant follicular stimulation hormone (rFSH; Gonal-F, Merck Serono, Darmstadt, Germany; Puregon, Merck Sharp & Dohme, Ravensburg, Germany; Jinsaiheng, Changchun GeneScience Pharmaceuticals, Changchun, China) was injected daily from days 2~3 of menstruation until the trigger day. When the leading follicle had reached 12~14 mm and/or serum level of LH was ≥5 mIU/mL, 0.25 mg/day of GnRH antagonist (Ganirelix, Merck Sharp & Dohme; Cetrorelix, MerckSerono) was administered until the trigger day. For the GnRH agonist protocol, 3.75 mg of leuprorelin acetate (Beiyi, Shanghai Livzon Pharmaceutical Co., Ltd., Zhuhai, Guangdong, China) was injected once on days 2~3 of menstruation. At least 28 days later, 100~300 IU/day of rFSH was injected until the trigger day. With both ovarian stimulation protocols, the initiation rFSH dose was determined by age, ovarian reserve, and BMI, and was adjusted according to the ovarian response. When the diameter of at least one or two follicles reached ≥18 mm, 250 μg of recombinant human chorionic gonadotrophin (Merck Serono) was injected, and transvaginal oocyte retrieval was performed 36 h later.

### *In vitro* fertilization and embryo culture

Following oocyte retrieval, the cumulus-oocyte complex was incubated at 37 °C in a 6% CO_2_ and 5% O_2_ environment. Approximately 3~4 h later, IVF was performed for all retrieved oocytes, while ICSI was performed for metaphase II oocytes (MII) after the granulosa cells were stripped off with an egg-stripping needle. An MII oocyte was defined as the oocyte with the first polar body discharged from the perivitelline gap. About 16~18 h after fertilization, a normal fertilized oocyte was defined as having two pronuclear (2PN) zygotes in the cytoplasm. Morphological grading of the day 3 cleavage embryo and blastocyst was carried out in accordance with the Istanbul consensus, and cleavage embryos of grade A~C and blastocysts of grade 4BC or higher were considered transplantable (Alpha Scientists in Reproductive Medicine and ESHRE Special Interest Group of Embryology, 2011). After fresh cleavage embryo transfer, should the number of high-quality cleavage embryos with eight cells exceed 4, all remaining cleavage embryos were cultured for blastocyst.

The MII rate referred to the number of MII oocytes divided by the number of oocytes retrieved. The 2PN rate referred to the number of 2PN divided by the number of oocytes fertilized with sperm. The cleavage embryo rate was calculated as the number of day 3 cleavage embryos divided by the number of cleaved embryos on day 2 (from 0 PN, 1PN, and 2PN). The blastocyst rate was calculated as the number of transplantable blastocysts divided by the number of day 3 cleavage embryos cultured for blastocyst.

### Fresh embryo transfer and luteinizing phase support

All embryos were transferred under trans-abdominal ultrasound guidance. In most cases, the two cleavage embryos with the highest morphological grade were transferred. Only one embryo was transferred under any of the following conditions: (1) only one transferable embryo had formed; (2) to avoid multiple pregnancy (scarred uterus, uterine malformation, cervical incompetence, body height <150 cm); and (3) by the request of the patient. When the criteria of single embryo transfer and blastocyst culture were both met, one blastocyst was transferred.

Luteal phase support was initiated on the day after oocyte retrieval by the injection of 60 mg/day progesterone oil (Zhejiang Xianju Pharmaceutical Co., Ltd. Taizhou, China) or vaginal progesterone (Crinone 8% gel, Merck, Germany), and oral administration of 20 mg/day of dydrogesterone (Duphaston, Abbott Healthcare Products B.V., The Netherlands).

### Outcomes of fresh embryo transfer

IR was calculated as the number of gestational sacs divided by the number of transferred embryos. CPR and LBR were calculated as the number of clinical pregnancy (presence of gestational sac by ultrasound examination) or live birth (delivery of a live fetus after 28 weeks of pregnancy) cycles divided by the number of embryo transfer cycles, respectively. The rates of multiple pregnancy, early miscarriage, and late miscarriage were calculated as the number of multiple pregnancy cycles, early miscarriage cycles (loss of pregnancy before 12 gestational weeks), and late miscarriage cycles (loss of pregnancy between 12 and 28 gestational weeks) divided by the number of clinical pregnancy cycles, respectively. Ovarian hyperstimulation syndrome was diagnosed and graded according to Navot, Bergh & Laufer (2019).

### Statistical analysis

The primary outcome was LBR. All analyses were performed using R-4.2.0 (R Foundation for Statistical Computing) software. Continuous variables were presented as mean ± standard deviation and were compared by *t*-tests. Categorical characteristics were presented as numbers and percentages and were analyzed using Chi-squared test or Fisher exact test. Missing values for key variables were imputed by predictive mean matching should they be <20%, or abandoned for further analysis should they exceeded 20% (Baugh et al., 2022; Heymans & Twisk, 2022). Propensity score matching (PSM) was performed to adjust the baseline characteristics of the ethnic Yi and Han groups using a 1:1 nearest-neighbor matching strategy without a caliper. Age, type and etiology of infertility, history of spontaneous abortion and tuberculosis, habit of smoking and alcohol, body mass index (BMI), protocol of OS, and fertilization were selected as the matching factors, as such factors have been reported as potential confounders that may affect the outcome of IVF-ET (Firns et al., 2015; Gai et al., 2024; Shingshetty et al., 2024; Templeton, Morris & Parslow, 1996). Standardized mean difference was estimated to evaluate the balance of baseline variables between the two groups before and after the matching. A standardized mean difference of <10.0% was regarded as a relatively small imbalance. Subgroup analyses were conducted to explore the potential differences of LBR across maternal age and BMI by multiple logistic regression. Since this was an exploratory investigation, the issue of multiple comparisons was not addressed through subgroup analysis. In addition, risk factors for LBR among ethnic Han and Yi women, including age, type and etiology of infertility, history of spontaneous abortion and tuberculosis infection, habit of smoking and alcohol use, BMI, and medical center, were respectively explored using a multiple regression model, and the potential risk factors were included. Two-tailed *P* values < 0.05 were considered statistically significant.

## Results

### Basal characteristics of the study population before and after matching

**Figure 1:**
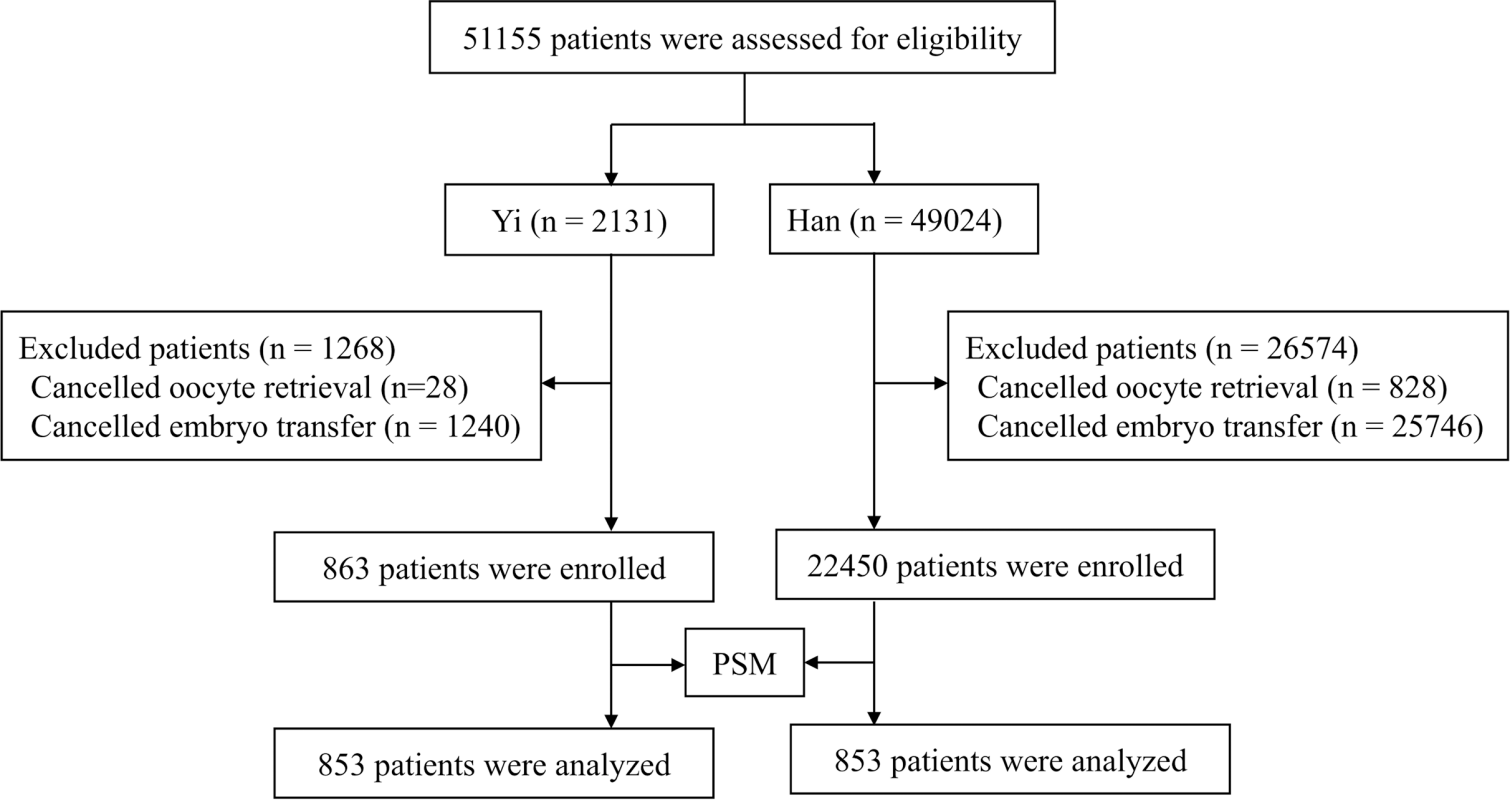
Flowchart of enrollment. Progress of retrospective cohort study including enrollment, exclusion, and completion.

A flowchart for patient enrollment is shown in Fig. 1. After excluding patients whose oocyte retrieval or embryo transfer was cancelled, 863 Yi and 22,450 Han women were enrolled. Basal FSH is missing in 178 cases, which accounted for 0.76% of all cases; Anti-Müllerian hormone (AMH) is missing in 46 cases, which accounted for 0.20% of all cases; high-quality blastocysts are missing in 20,205 cases, which accounted for 86.66% of all cases. Therefore, multiple imputation was used for missing values of basal FSH and AMH, while variables for high-quality blastocysts was not analyzed. Compared to ethnic Hans, ethnic Yis had a higher age and basal FSH, greater history of tuberculosis infection, increased habits of smoking and alcohol use, higher BMI, and lower history of spontaneous abortion and AMH (*P* < 0.05). The type and etiology of infertility and protocols of ovarian stimulation and fertilization were also significantly different between the two groups (*P* < 0.05). After PSM, the above basal characteristics were not significantly different between the two groups (*P* > 0.05; Table 1).

**Table 1:** Basal characteristics of the study population before and after matching.

Characteristics	Before matching				After matching			
	Yi (*n* = 863)	Han (*n* = 22,450)	SMD	*P* value	Yi (*n* = 853)	Han (*n* = 853)	SMD	*P* value
Age (year)	31.67 ± 4.70	30.91 ± 4.04	0.161	<0.001	31.66 ± 4.72	31.72 ± 4.26	0.012	0.804
AMH (ng/mL)	3.07 ± 2.05	3.55 ± 2.39	0.215	<0.001	3.07 ± 2.06	3.07 ± 2.10	0.001	0.984
Basal FSH (IU/L)	7.65 ± 2.45	7.49 ± 2.16	0.070	0.031	7.65 ± 2.46	7.72 ± 2.49	0.027	0.574
Type of infertility (%)			0.040	<0.001			0.021	0.698
Primary	50.17% (433/863)	46.17% (10,365/22,450)			48.77% (416/853)	49.82% (425/853)		
Secondary	49.83% (430/863)	53.83% (12,085/22,450)			51.23% (437/853)	50.64% (428/853)		
Etiology of infertility (%)			0.016	<0.001			0.055	0.938
Tubal factor	30.82% (266/863)	58.16% (13,057/22,450)			29.31% (250/853)	31.18% (266/853)		
Ovulation disorder	1.39% (12/863)	3.20% (718/22,450)			1.41% (12/853)	1.64% (14/853)		
Endometriosis	0.23% (2/863)	2.11% (474/22,450)			0.23% (2/853)	0.23% (2/853)		
Male factor	10.54% (91/863)	10.41% (2,337/22,450)			10.55% (90/853)	10.55% (90/853)		
Mixed factors	54.58% (471/863)	22.83% (5,125/22,450)			55.22% (471/853)	54.16% (462/853)		
Unexplained infertility	2.43% (21/863)	3.29% (739/22,450)			2.46% (21/853)	3.04% (26/853)		
History of spontaneous abortion (%)	5.21% (45/863)	12.46% (2,798/22,450)	0.072	<0.001	5.27% (45/853)	5.51% (47/853)	0.010	0.915
History of tuberculosis infection (%)	7.30% (63/863)	2.20% (494/22,450)	0.051	<0.001	6.33% (54/853)	6.68% (57/853)	0.014	0.844
Smoking (%)	6.72% (58/863)	3.54% (795/22,450)	0.032	<0.001	6.80% (58/853)	6.10% (52/853)	0.029	0.622
Alcohol (%)	0.81% (7/863)	0.36% (81/22,450)	0.005	<0.001	0.82% (7/853)	0.94% (8/853)	0.013	1.000
BMI (kg/m^2^)	23.37 ± 3.05	21.99 ± 3.03	0.452	<0.001	23.33 ± 3.03	23.19 ± 3.51	0.042	0.390
OS protocol (%)			0.069	<0.001			0.023	0.679
GnRH agonist	67.21% (580/863)	74.09% (16,633/22,450)			67.17% (573/853)	68.23% (582/853)		
GnRH antagonist	32.79% (283/863)	25.91% (5,817/22,450)			32.83% (280/853)	31.77% (271/853)		
Fertilization protocol (%)			0.050	<0.001			0.006	0.948
IVF	83.43% (720/863)	88.38% (19,842/22,450)			83.47% (712/853)	83.70% (714/853)		
ICSI	16.57% (143/863)	11.62% (2,608/22,450)			16.53% (141/853)	16.30% (139/853)		

**Note:**

AMH, Anti-Müllerian hormone; FSH, follicular stimulating hormone; BMI, body mass index; OS, ovarian stimulation; GnRH, gonadotropin-releasing hormone; IVF, *in vitro* fertilization; ICSI, intracytoplasmic sperm injection.

### Outcomes of ovarian stimulation and IVF-ET after PSM

Compared to ethnic Hans, ethnic Yis had shorter rFSH duration, higher serum level of E_2_ on the trigger day, and more oocytes retrieved, and lower IR, CPR, ongoing pregnancy rate, and LBR (*P* < 0.05). The rFSH dose, serum level of P on the trigger day, cleavage embryo rate, endometrial thickness, type and number of embryos transferred, gestational age at delivery, birth weight, rates of moderate and severe ovarian hyperstimulation syndrome, MII, 2PN, blastocyst, early and late miscarriage, and ectopic and multiple pregnancy rates were not significantly different between the two groups (*P* > 0.05; Table 2).

**Table 2:** Controlled ovarian stimulation and outcomes *in vitro* fertilization-fresh embryo transfer.

Variable	Yi (*n* = 853)	Han (*n* = 853)	Difference (95% CI)	*P* value
rFSH dose (IU)	2,129.09 ± 692.78	2,174.70 ± 643.47	45.61 [−17.89 to 109.11]	0.159
rFSH duration (day)	10.36 ± 2.14	10.63 ± 2.21	0.27 [0.07 to 0.48]	0.010
E_2_ on the trigger day (pg/mL)	2,466.37 ± 1,297.09	2,264.02 ± 1,296.17	−202.35 [−325.93 to −78.77]	0.001
P on the trigger day (ng/mL)	0.89 ± 0.64	0.86 ± 0.44	−0.03 [−0.09 to 0.02]	0.202
Oocytes retrieved	9.66 ± 4.66	9.05 ± 4.45	−0.61 [−0.10 to −0.18]	0.006
Moderate and severe OHSS rate (%)	1.41% (12/853)	1.76% (15/853)	−0.35% [−1.54% to 0.83%]	0.561
MII rate (%)	87.60% (7,220/8,242)	88.12% (6,803/7,720)	0.52% [−1.54% to 0.49%]	0.313
2PN rate (%)	65.92% (5,189/7,970)	65.11% (4,939/7,492)	−0.82% [−2.32% to 0.68%]	0.285
Cleavage embryo rate (%)	81.92% (6,529/7,970)	81.78% (6,127/7,492)	0.14% [−1.08% to 1.35%]	0.822
Blastocyst rate (%)	58.05% (2,157/3,716)	56.91% (2,034/3,574)	−1.14% [−3.41 to 1.13%]	0.327
Endometrial thickness (mm)	10.71 ± 2.41	10.71 ± 2.41	−0.61 [−0.24 to 0.20]	0.881
Type of embryo transferred (%)				0.414
Cleavage embryo	78.66% (671/853)	77.02% (657/853)		
Blastocyst	21.34% (182/853)	22.98% (196/853)		
Number of embryos transferred (%)				0.235
One	17.27% (147/853)	19.46% (166/853)		
Two	82.73% (706/853)	80.54% (687/853)		
IR (%)	32.14% (501/1559)	36.84% (561/1523)	−4.71% [−8.05% to −1.35%]	0.006
CPR (%)	45.37% (387/853)	51.35% (438/853)	−5.98% [−10.71% to −1.24%]	0.013
Early miscarriage rate (%)	14.99% (58/387)	15.30% (67/438)	−0.31% [−5.21% to 4.59%]	0.901
Late miscarriage rate (%)	3.10% (12/387)	1.60% (7/438)	1.50% [−0.59% to 3.59%]	0.151
Ectopic pregnancy rate (%)	2.84% (11/387)	1.60% (7/438)	1.24% [0.79 to 3.27%]	0.222
Ongoing pregnancy rate (%)	37.05% (316/853)	42.67% (364/853)	5.63% [−10.27% to −0.99%]	0.018
Multiple pregnancy rate (%)	29.46% (114/387)	28.02% (123/438)	1.38% [4.82% to 7.57%]	0.663
LBR (%)	35.64% (304/853)	41.74% (356/853)	−6.10% [−10.71% to −1.48%]	0.010
Gestational age at delivery (week)	38.12 ± 2.11	37.92 ± 2.12	0.17 [−0.53 to 0.12]	0.213
Birth weight (g)	3,019.40 ± 581.50	3,041.70 ± 634.70	−22.31 [−71.17 to 115.79]	0.640

**Note:**

rFSH, recombinant follicular stimulation hormone; E_2_, estradiol; P, progesterone; OHSS, Ovarian hyper-stimulation syndrome; 2PN, two pronuclear; IR, implantation rate; CPR, clinical pregnancy rate; LBR, live birth rate.

After PSM, basal FSH, protocol of OS and multiple pregnancy were significantly different (*P* < 0.05), while ethnicity, AMH, protocol of fertilization, IR, CPR, LBR, and rates of early and late miscarriage, ectopic pregnancy, and ongoing pregnancy were not significantly different between patients from the three centers (*P* > 0.05; Table S1).

For patients who achieved live birth, the age, AMH, basal FSH, type and etiology of infertility, history of spontaneous abortion or tuberculosis infection, habit of smoking or alcohol use, BMI, and protocol of ovarian stimulation and fertilization were not significantly different between the two groups (*P* > 0.05; Table S2).

There were 12 and eight stillbirths, 304 and 356 live-born infants, and three and five birth defects among the ethnic Yi and Han women, respectively. The birth defects included congenital heart defect, hearing disorder, trisomy 21, trisomy 12, and micromandibulo-sagging tongue syndrome. The mean gestational age at delivery was 38 weeks (range: 28~42 weeks) for ethnic Yis and 38 weeks (range: 28~42 weeks) for ethnic Hans. The mean birth weight was 2,912 g (range: 450~4,940 g) for ethnic Yis and 2,922 g (range: 1,000~6,560 g) for ethnic Hans.

### Regression analysis of the risk factors for LBR among Yi and Han women

Of the 863 ethnic Yi women and 22,450 ethnic Han women, multiple logistic regression analysis showed maternal age as an important factor for LBR in both Yi (adjusted OR = 0.93, 95% CI [0.90-0.96], *P* < 0.001) and Han (adjusted OR = 0.96, 95% CI [0.95–0.96], *P* < 0.001) women. For ethnic Han women, an overweight BMI presented as a risk factor for a lower LBR compared to those with normal BMIs (adjusted OR = 0.92, 95% CI [0.86–0.98], *P* = 0.008), and male-factor infertility presented as a slightly protective factor for live birth compared to other infertile factors (adjusted OR = 1.10, 95% CI [1.00–1.20], *P* = 0.046). No significant association was found between LBR and other factors in both groups, including AMH, basal FSH, the type and etiology of infertility, history of spontaneous abortion and tuberculosis infection, smoking, alcohol use, and medical center (Table 3).

**Table 3:** Risk factors for live birth rate of Yi or Han women.

Characteristics	Yi women (*n* = 863)		Han women (*n* = 22,450)	
	Adjusted *OR* (95% CI)	*P* value	Adjusted *OR* (95% CI)	*P* value
Age	0.93 [0.90–0.96]	<0.001	0.96 [0.95–0.96]	<0.001
AMH	0.95 [0.88–1.03]	0.220	1.00 [0.99–1.01]	0.849
Basal FSH	1.00 [0.94–1.06]	0.942	1.00 [0.99–1.02]	0.806
Type of infertility (Reference: Primary infertility)				
Secondary infertility	1.32 [0.97–1.79]	0.075	1.01 [0.95–1.07]	0.799
Etiology of infertility (Reference: Tubal factors)				
Ovulatory disorder	1.32 [0.39–4.48]	0.657	1.09 [0.93–1.27]	0.302
Endometriosis	0 [0–Inf]	0.999	1.07 [0.89–1.29]	0.457
Male factors	0.94 [0.55–1.59]	0.804	1.10 [1.00–1.20]	0.046
Mixed factors	1.05 [0.75–1.48]	0.767	1.06 [0.99–1.13]	0.105
Unexplained infertility	0.86 [0.33–2.27]	0.760	0.97 [0.83–1.13]	0.720
History of spontaneous abortion	1.50 [0.77–2.89]	0.232	1.02 [0.94–1.11]	0.686
History of tuberculosis infection	1.02 [0.59–1.75]	0.948	0.96 [0.80–1.15]	0.621
Smoking	1.00 [0.56–1.78]	0.989	1.10 [0.95–1.27]	0.205
Alcohol	2.66 [0.57–12.40]	0.213	0.75 [0.47–1.20]	0.228
BMI (Reference: 18.5–24)				
BMI < 18.5	0.81 [0.39–1.67]	0.564	1.03 [0.94–1.13]	0.571
BMI > 24	0.89 [0.66–1.21]	0.459	0.92 [0.86–0.98]	0.008
Center (Reference: Center one)				
Center two	1.16 [0.81–1.65]	0.423	1.01 [0.91–1.12]	0.840
Center three	0.70 [0.45–1.10]	0.122	0.91 [0.82–1.00]	0.058

**Note:**

AMH, Anti-Müllerian hormone; FSH, follicular stimulating hormone; BMI, body mass index.

### Subgroups analysis of LBR in the matched cohort

**Figure 2:**
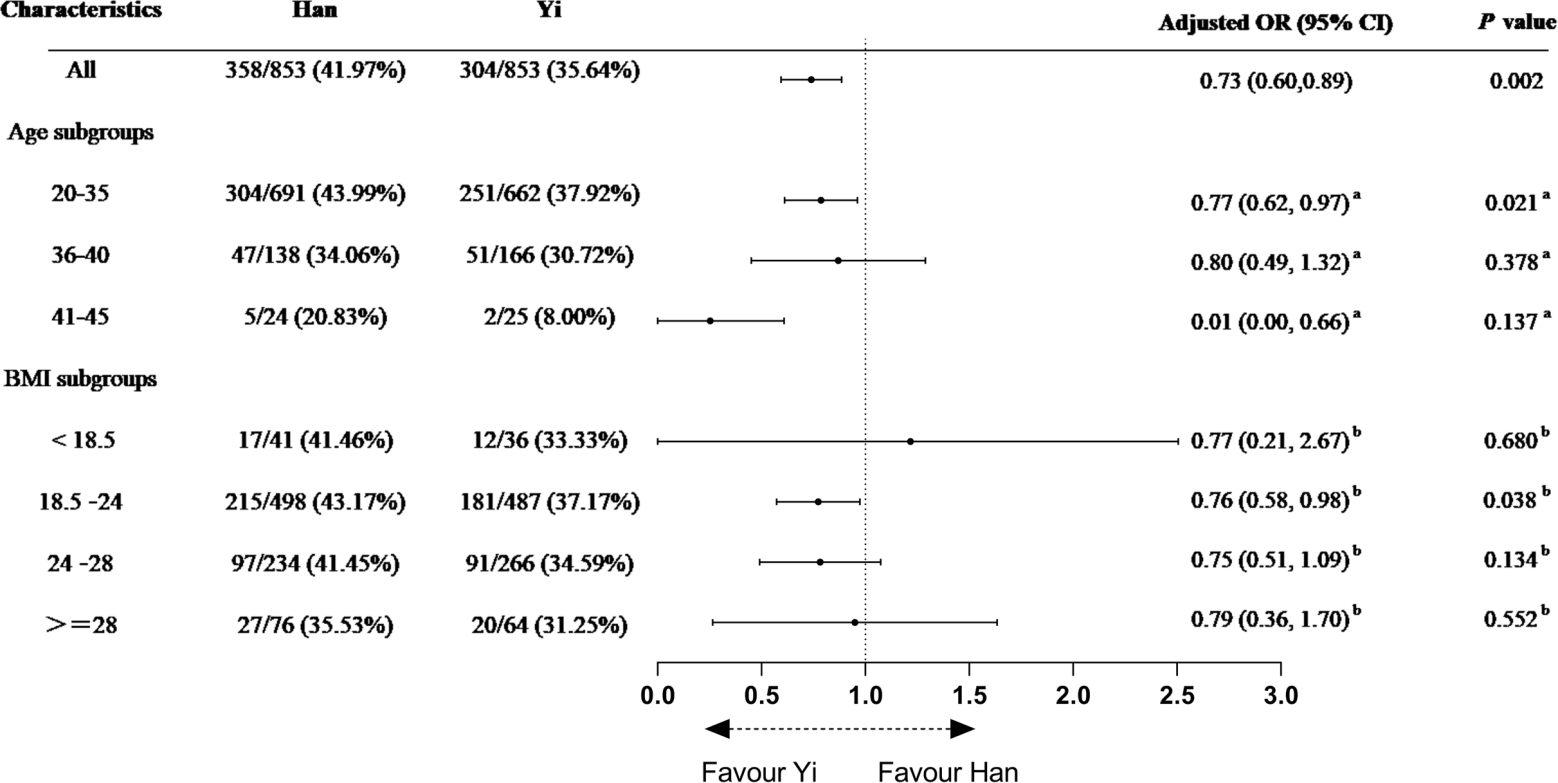
Subgroup analysis of live birth rate in the matched cohort. Age and BMI subgroup analysis of live birth rate in the matched cohort. AMH, Anti-Müllerian hormone; FSH, follicular stimulating hormone; BMI, body mass index; OR, odds ratio. Yi women were the reference. a Adjusted for BMI, AMH, basal FSH, type and etiology of infertility, history of spontaneous abortion and tuberculosis, habit of smoking and alcohol, protocol of OS and fertilization. b Adjusted for age, AMH, basal FSH, type and etiology of infertility, history of spontaneous abortion and tuberculosis, habit of smoking and alcohol, protocol of OS and fertilization.

After adjusting for potential confounding variables, LBR of ethnic Han women was significantly higher than that of ethnic Yi women (adjusted OR = 0.73, 95% CI [0.60–0.89], *P* = 0.002). Age subgroup analysis showed that LBR was significantly different between Yi and Han women of a younger age (20~35 years old; adjusted OR = 0.77, 95% CI [0.62–0.97], *P* = 0.021). BMI subgroup analysis indicated that LBR was significantly different between ethnic Yi and Han women with a normal BMI (18.5~24.0 kg/m^2^; adjusted OR = 0.76, 95% CI [0.58–0.98], *P* = 0.038; Fig. 2).

## Discussion

This study found that the IR, CPR, ongoing pregnancy rate, and LBR was lower among ethnic Yi women compared to ethnic Han women after adjusting for basal characteristics by PSM. From subgroups analysis, the LBR of ethnic Han women was higher than that of Yi women. Furthermore, age was an important factor for LBR in both Yi and Han women, while BMI was an important factor for LBR among overweight Han women.

For both Yi and Han women, the difference of living environment, socioeconomic factors such as dietary and household income, and genetic factors may account for the disparities in LBR. First, ethnic Yis mainly live in plateaus, while ethnic Hans mainly live in plains. The hypobaric hypoxia at high altitude may affect the reproductive function in females though multiple mechanisms, including disturbed hypothalamus-pituitary-ovary axis, decreased follicular reserve, delayed embryonic development, and increased embryonic mortality (Zhong et al., 2024; Parraguez & Gonzalez-Bulnes, 2020). Bangham & Sacherer (1980) found that the overall fertility rate of Sherpas living at high altitudes was lower than those living at low altitudes (4.77 *vs*. 7.7). Second, the main diet for ethnic Yis is less healthy, with an energy supply mainly derived from plants (cereal and potatoes) and high fat (processed pork meat), with insufficient intake of protein, unsaturated fat, and calcium (Zhou et al., 2003). A high-fat diet may have a negative effect on ovulation and embryo implantation by inflammation and oxidative stress, as evidenced by increased inflammatory mediators, reactive oxygen species induced apoptosis and lipid peroxidation in ovaries, and altered expression of genes involved in such pathways (Berardino et al., 2024; Gonnella et al., 2022). Hartman et al. (2021) found that a diet with high energy and saturated fats, and with low amounts of fruits, vegetables, and grains, was associated with a 70% reduction in CPR (OR = 0.30, 95% CI [0.11–0.81]) when comparing the highest to the middle adherence tertile. Third, poor access to healthcare due to low income may also contribute to lower LBR. Manelyan et al. (2023) reported that patients living in neighborhoods with a lower annual household income had a lower LBR compared with those living in more affluent areas. Zhang (2017) reported that the income of ethnic Hans was much higher than that of ethnic Yis. Fourth, ethnic difference in ovarian function may also affect the outcomes of IVF-ET. A prospective study has found that serum level of AMH may vary between different ethnicities in Asia (Tzeng et al., 2023). Purcell et al. (2007) showed that, compared to Caucasians, Asian women undergoing IVF achieved significantly fewer pregnancies, which may be attributable to differences in ovarian reserve (Gleicher et al., 2007). Hypobaric hypoxia caused by high altitude and an unhealthy diet may also contribute to the poor ovarian function of ethnic Yis in this study. Taken together, geographical, socioeconomic, dietary, and genetic factors may have collectively contributed to the disparities in the outcome of IVF-ET (Seifer, Sharara & Jain, 2022). Due to the retrospective nature of this study, the difference in LBR between ethnic Han and Yi women should be interpreted with caution.

This study also found age as an important factor for LBR in both ethnic Yi and Han women, with younger women attaining a higher LBR. As reported by many studies, age is the most reliable predictor for the success of IVF-ET (Shingshetty et al., 2024; ART success rates by clinic from Centers for Disease Control and Prevention, 2022). The age-related female fertility decline is primarily due to the reduction in quality and quantity of oocytes (Wang et al., 2025). Many molecular changes, including genomic instability, mitochondrial impairment, oxidative stress, apoptosis, cellular senescence, and inflammation have been associated with ovarian aging (Wang et al., 2024). In this study, age-subgroup analysis indicated that the difference in LBR between ethnic Yis and Hans was most pronounced at 20~35 years, though this may also be attributable to the large sample size of the younger-age group. Another reason may be that the difference in ovarian reserve between ethnic Yi and Han women was most pronounced at a younger age, but may diminish over time, resulting in similar LBR between older women.

Higher BMI may be another risk factor for the adverse outcomes of IVF-ET (Sermondade et al., 2019). Provost et al. (2016) analyzed 239,127 IVF cycles and found IR, CPR, and LBR to be higher in women with low and normal BMIs, which had declined with increasing BMI. In the current study, LBR was lower among overweight Yi and Han women (BMI > 24.0 kg/m^2^) compared to those with a normal weight, and this was more pronounced among ethnic Han women. Therefore, maintaining a normal BMI may be beneficial for LBR, especially in Han women. Of note, the LBR was lower in Yi women compared to Han women with a normal BMI, while no significant difference was found between thin, overweight, and obese women. Considering the large sample size for women with a normal BMI, the much smaller sample sizes for thin, overweight, and obese women may account for the phenomenon. Luke et al. (2011) also found that LBR was lower in normal-weight Black women compared to normal weight White women. Therefore, the disparity of the LBR between ethnic Yi and Han women with a normal BMIs may be attributed to their genetic backgrounds and environmental factors.

### Strengths and limitations

The multicenter study was the first to explore the difference in the outcomes of IVF-ET among different Chinese ethnic groups with a large sample size. The limitations of this study include: First, the inherent nature of multi-center and retrospective studies may introduce a selection bias; Second, the information for ovarian reserve indicators was incomplete in few patients, and the information for high-quality blastocysts was incomplete in many patients; Third, social determinants of health and genetic validation for ethnicity were lacking; Fourth, the information of FET cycle and LBR per oocyte retrieval cycle and per patient at the three centers were unavailable. Therefore, in the absence of detailed data on socioeconomic status, lifestyle, and high-quality blastocysts, ethnicity should be interpreted as a surrogate marker for these complex, unmeasured environmental and social contexts, rather than a direct biological determinant.

## Conclusions

In summary, this multi-center retrospective study with a large sample size showed that the outcomes of IVF-ET were different between ethnic Yi and Han women, and that ethnicity may act as a proxy influencing LBR. The observed ethnic disparities may be attributed to underlying differences of complex factors. Maintaining a normal BMI before undergoing IVF-ET was beneficial for both ethnic Yi and Han women.

## Supplemental Information

10.7717/peerj.21145/supp-1 Supplemental Information 1 Raw data.

10.7717/peerj.21145/supp-2 Supplemental Information 2 Ethnic composition of patients and outcomes of IVF-ET at the different centers after matching. Notes: AMH, Anti-M ü llerian Hormone; FSH, follicle stimulating hormone; OS, ovarian stimulation; GnRH, gonadotropin-releasing hormone; IVF, *in vitro* fertilization; ICSI, intracytoplasmic sperm injection; IR, implantation rate; CPR, clinical pregnancy rate; LBR, live birth rate

10.7717/peerj.21145/supp-3 Supplemental Information 3 Characteristics of Yi or Han women patients who achieved live birth. Note: AMH, Anti-M ü llerian Hormone; FSH, follicle stimulating hormone; BMI, body mass index ; OS, ovarian stimulation; GnRH, gonadotropin-releasing hormone; IVF, *in vitro* fertilization; ICSI, intracytoplasmic sperm injection.

10.7717/peerj.21145/supp-4 Supplemental Information 4 STROBE Checklist.

## Additional Information and Declarations

### Data Availability

The following information was supplied regarding data availability:

The raw data is available in the Supplemental File.

## References

[ref-1] Alpha Scientists in Reproductive Medicine and ESHRE Special Interest Group of Embryology (2011). The Istanbul consensus workshop on embryo assessment: proceedings of an expert meeting. Human Reproduction.

[ref-2] Bangham CR, Sacherer JM (1980). Fertility of Nepalese Sherpas at moderate altitude: comparison with high-altitude data. Annual Human Biology.

[ref-3] Baugh A, Buhr RG, Quibrera P, Barjaktarevic I, Barr RG, Bowler R, Han MK, Kaufman JD, Koch AL, Krishnan J, Labaki W, Martinez FJ, Mkorombindo T, Namen A, Ortega V, Paine R, Peters SP, Schotland H, Sundar K, Zeidler MR, Hansel NN, Woodruff PG, Thakur N (2022). Risk of COPD exacerbation is increased by poor sleep quality and modified by social adversity. Sleep.

[ref-4] Bendikson K, Cramer DW, Vitonis A, Hornstein MD (2005). Ethnic background and in vitro fertilization outcomes. International Journal of Gynaecology and Obstetrics.

[ref-5] Berardino CD, Barceviciute U, Rapini CCS, Peserico A, Capacchietti G, Bernabo N, Russo V, Gatta V, Konstantinidou F, Donato M, Barboni B (2024). High-fat diet-negative impact on female fertility: from mechanisms to protective actions of antioxidant matrices. Frontiers in Nutrition.

[ref-6] Beroukhim G, Seifer DB (2023). Racial and ethnic disparities in access to and outcomes of infertility treatment and assisted reproductive technology in the United States. Endocrinology and Metabolism Clinics of North America.

[ref-7] Boivin J, Bunting L, Collins JA, Nygren KG (2007). International estimates of infertility prevalence and treatment-seeking: potential need and demand for infertility medical care. Human Reproduction.

[ref-8] Centers for Disease Control and Prevention (2022). ART success rates by clinic. http://nccd.cdc.gov/drh_art.

[ref-9] Firns S, Cruzat FC, Keane KN, Joesbury KA, Lee AH, Newsholme P, Yovich JL (2015). The effect of cigarette smoking, alcohol consumption and fruit and vegetable consumption on IVF outcomes: a review and presentation of original data. Reproductive Biology and Endocrinology.

[ref-10] Gai XY, Chi HB, Li R, Sun YC (2024). Tuberculosis in infertility and in vitro fertilization-embryo transfer. Chinese Medical Journal.

[ref-11] Gleicher N, Weghofer A, Li J, Barad D (2007). Differences in ovarian function parameters between Chinese and Caucasian oocyte donors: do they offer an explanation for lower IVF pregnancy rates in Chinese women?. Human Reproduction.

[ref-12] Gonnella F, Konstantinidou F, Berardino CD, Capacchietti G, Peserico A, Russo V, Barboni B, Stuppia L, Gatta V (2022). A systematic review of the effects of high-fat diet exposure on oocyte and follicular quality: a molecular point of view. International Journal of Molecular Science.

[ref-13] Hartman TJ, Fung JL, Hsiao PY, Fan W, Mitchell DC, Goldman MB (2021). Dietary energy density and fertility: results from the lifestyle and fertility study. Current Developments in Nutrition.

[ref-14] Heymans MW, Twisk JWR (2022). Handling missing data in clinical research. Journal of Clinical Epidemiology.

[ref-15] Huang Y (2023). Comparison of pregnancy outcomes of patients with polycystic ovary syndrome using GnRH-a long-acting regimen in Guangxi Zhuang and Han. Master’s Thesis of Guang Xi Medical University.

[ref-16] Langen ES, Shahine LK, Lamb JD, Lathi RB, Milki AA, Fujimoto VY, Westphal LM (2010). Asian ethnicity and poor outcomes after in vitro fertilization blastocyst transfer. Obstetrics and Gynecology.

[ref-17] Lee IT, Berger DS, Koelper N, Senapati S, Mainigi M (2023). Race, ovarian responsiveness, and live birth following in vitro fertilization. Fertility and Sterility.

[ref-18] Lin JL, Zhang LH (2018). The effect of ethnic differences on assisted reproductive treatment outcomes: a single-center retrospective analysis. Journal of Reproductive Medicine.

[ref-19] Luke B, Brown MB, Stern JE, Missmer SA, Fujimoto VY, Leach R (2011). Racial and ethnic disparities in assisted reproductive technology pregnancy and live birth rates within body mass index categories. Fertility and Sterility.

[ref-20] Manelyan E, Abittan B, Shan WW, Shahani D, Kwait B, Rausch M, Blitz MJ (2023). Socioeconomic disparities in fertility treatments and associated likelihood of livebirth following in vitro fertilization. Archives of Gynecology and Obstetric.

[ref-21] Navot D, Bergh PA, Laufer N (2019). Ovarian hyperstimulation syndrome in novel reproductive technologies: prevention and treatment. Fertility and Sterility.

[ref-22] Office of the Seventh National Census Leading Group of The State Council (2022). China population census yearbook 2020.

[ref-23] Parraguez VH, Gonzalez-Bulnes A (2020). Endocrinology of reproductive function and pregnancy at high altitudes. Current Opinion in Endocrine and Metabolic Research.

[ref-24] Provost MP, Acharya KS, Acharya CR, Yeh JS, Steward RG, Eaton JL, Goldfarb JM, Muasher SJ (2016). Pregnancy outcomes decline with increasing body mass index: analysis of 239,127 fresh autologous in vitro fertilization cycles from the 2008–2010 society for assisted reproductive technology registry. Fertility and Sterility.

[ref-25] Purcell K, Schembri M, Frazier LM, Rall MJ, Shen SH, Croughan M, Grainger DA, Fujimoto VY (2007). Asian ethnicity is associated with reduced pregnancy outcomes after assisted reproductive technology. Fertility and Sterility.

[ref-26] Seifer DB, Sharara FI, Jain T (2022). The disparities in ART (DART) hypothesis racial and ethnic disparities in access and outcomes of IVF treatment in the USA. Reproductive Science.

[ref-27] Sermondade N, Huberlant S, Bourhis-LefeBvre C, Arbo E, Gollot V, Colombani M, Freour T (2019). Female obesity is negatively associated with live birth rate following IVF: a systematic review and meta-analysis. Human Reproduction Update.

[ref-28] Sharara FI, McClamrock HD (2000). Differences in in vitro fertilization (IVF) outcome between white and black women in an inner-city, university-based IVF program. Fertility and Sterility.

[ref-29] Shingshetty L, Cameron NJ, Mclernon DJ, Bhattacharya S (2024). Predictors of success following IVF. Fertility and Sterility.

[ref-30] Templeton A, Morris JK, Parslow W (1996). Factors that affect outcome of in-vitro fertilization treatment. The Lancet.

[ref-31] Tzeng CR, Huang ZW, Asada Y, Zhang CL, Ho MT, Li RHW, Kim JH, Govindarajan M, Vuyavanich T, Sini I, Wong PS, Singh S, Lin WY, Ho NT (2023). Factors affecting the distribution of serum anti-Müllerian hormone levels among infertile Asian women: a multi-nation, multi-centre, and multi-ethnicity prospective cohort study. Human Reproduction.

[ref-32] Wang NF, Mamsen LS, Cadenas J, Saritas G, Machlon KT, Fedder J, Ernst E, Johannsen ML, Kristensen SG, Kelsey T, Kumar A, Kalra B, Lossl K, Anderson CY (2025). Impact of female age on concentrations of reproductive hormones and oocyte-specific growth factors in follicular fluid from human small antral follicles. Human Reproduction.

[ref-33] Wang S, Ren J, Jing Y, Qu J, Liu GH (2024). Perspectives on biomarkers of reproductive aging for fertility and beyond. Nature Aging.

[ref-34] Zhang JH (2017). A study on the ethnic gap of income distribution of ethnic minorities. Guizhou Ethnic Studies.

[ref-35] Zhang YB, Zhao J, Ailun GW, Du C, Liu F, He J, Dai Bo, Ma X (2023). Comparison of main clinical and laboratory indicators in Mongolian and Han Chinese women treated with IVF-ET. Journal of Inner Mongolia Medical University.

[ref-36] Zhong Y, Liu FF, Zhang XJ, Guo QW, Wang ZH, Wang R (2024). Research progress on reproductive system damage caused by high altitude hypoxia. Endocrine.

[ref-37] Zhou JC, Huang CY, Xu YC, Sun GJ, Li XP, Pu JH, Yang XG (2003). The dietary patterns and nutritional status of adult Yi people in Liangshan autonomous region. Journal of Hygiene Research.

