# Supplementary material for: Comparison of the outcomes of in vitro fertilization and embryo transfer among ethnic Chinese Yi and Han women: a multicenter retrospective cohort study
Source: PeerJ. 2026 Apr 17;14:e21145. doi: 10.7717/peerj.21145 (PMC13094553; doi:10.7717/peerj.21145)
Supplement: Supplemental Information 2 — Notes: AMH, Anti-M ü llerian Hormone; FSH, follicle stimulating hormone; OS, ovarian stimulation; GnRH, gonadotropin-releasing hormone; IVF, in vitro fertilization; ICSI, intracytoplasmic sperm injection; IR, implantation rate; CPR, clinical pregnancy rate; LBR, live birth rate [file peerj-14-21145-s002.docx]

**Supplementary Table 1** **Ethnic composition of patients and outcomes of IVF-ET at the different centers after matching**

| **Characteristics** | **Center One (n = 561)** | **Center Two (n = 577)** | **Center Three (n = 568)** | ***p* value** |
| --- | --- | --- | --- | --- |
| Ethnic composition (%) |  |  |  | 0.917 |
| Yi | 50.4% (283/561) | 50.3% (290/577) | 49.3% (280/568) |  |
| Han | 49.6% (278/561) | 49.7% (287/577) | 50.7% (288/568) |  |
| AMH (ng/mL) | 3.06±1.85 | 3.09±2.11 | 3.05±2.26 | 0.949 |
| Basal FSH (IU/L) | 7.98±2.47 | 7.51±2.45 | 7.56±2.47 | 0.002 |
| OS protocol (%) |  |  |  | < 0.001 |
| GnRH agonist | 65.4% (367/561) | 77.1% (445/577) | 60.4% (343/568) |  |
| GnRH antagonist | 34.6% (194/561) | 22.9% (132/577) | 39.6% (225/568) |  |
| Fertilization protocol (%) |  |  |  | 0.277 |
| IVF | 81.6% (458/561) | 85.1% (491/577) | 84.0% (477/568) |  |
| ICSI | 18.4% (103/561) | 14.9% (86/577) | 16.0% (91/568) |  |
| IR (%) | 36.6% (358/979) | 34.6% (368/1065) | 31.8% (336/1055) | 0.079 |
| CPR (%) | 51.5% (289/561) | 47.3% (273/577) | 46.3% (263/568) | 0.178 |
| Early miscarriage rate (%) | 16.960% (49/289) | 14.3% (39/273) | 14.1% (37/263) | 0.568 |
| Late miscarriage rate (%) | 3.1% (9/289) | 1.5% (4/273) | 2.3% (6/263) | 0.428 |
| Ectopic pregnancy rate (%) | 2.4% (7/289) | 2.2% (6/273) | 1.9% (5/263) | 0.916 |
| Ongoing pregnancy rate (%) | 41.5% (233/561) | 39.5% (228/577) | 38.6% (219/568) | 0.581 |
| Multiple pregnancy rate (%) | 23.9% (69/289) | 34.8% (95/273) | 27.8% (73/263) | 0.015 |
| LBR (%) | 39.9% (224/561) | 38.5% (222/577) | 37.7% (214/568) | 0.733 |

**Notes:** AMH, Anti-Müllerian Hormone; FSH, follicle stimulating hormone; OS, ovarian stimulation; GnRH, gonadotropin-releasing hormone; IVF, in vitro fertilization; ICSI, intracytoplasmic sperm injection; IR, implantation rate; CPR, clinical pregnancy rate; LBR, live birth rate.
