# Supplementary material for: Comparison of the outcomes of in vitro fertilization and embryo transfer among ethnic Chinese Yi and Han women: a multicenter retrospective cohort study
Source: PeerJ. 2026 Apr 17;14:e21145. doi: 10.7717/peerj.21145 (PMC13094553; doi:10.7717/peerj.21145)
Supplement: Supplemental Information 3 — Note: AMH, Anti-M ü llerian Hormone; FSH, follicle stimulating hormone; BMI, body mass index ; OS, ovarian stimulation; GnRH, gonadotropin-releasing hormone; IVF, in vitro fertilization; ICSI, intracytoplasmic sperm injection. [file peerj-14-21145-s003.docx]

**Supplementary Table 2** **Characteristics of Yi or Han women patients who achieved live birth**

| **Characteristics** | **Yi (n=304)** | **Han (n=356)** | ***p* value** |
| --- | --- | --- | --- |
| Age (year) | 30.79 ± 4.41 | 31.22 ± 3.92 | 0.180 |
| AMH (ng/mL) | 3.09±1.83 | 3.18±2.03 | 0.550 |
| Basal FSH (IU/L) | 7.63±2.26 | 7.53±2.27 | 0.584 |
| Type of infertility (%) |  |  | 0.799 |
| Primary | 47.0% (143/304) | 48.0% (171/356) |  |
| Secondary | 53.0% (161/304) | 52.0% (185/356) |  |
| Etiology of infertility (%) |  |  | 0.727 |
| Tubal factor | 30.6% (93/304) | 33.7% (120/356) |  |
| Ovulation disorder | 1.6% (5/304) | 2.0% (7/356) |  |
| Endometriosis | 0% (0/304) | 0% (0/356) |  |
| Male factor | 8.9% (27/304) | 10.4% (37/356) |  |
| Mixed factors | 56.6% (172/304) | 51.1% (182/356) |  |
| Unexplained infertility | 2.3% (7/304) | 2.8% (10/356) |  |
| History of spontaneous abortion (%) | 6.3% (19/304) | 4.2% (15/356) | 0.238 |
| History of tuberculosis infection (%) | 6.9% (21/304) | 5.9% (21/356) | 0.597 |
| Smoking (%) | 6.6% (20/304) | 5.1% (18/356) | 0.403 |
| Alcohol (%) | 1.3% (4/304) | 1.1% (4/356) | 0.822 |
| BMI (kg/m^2^) | 23.22 ± 3.00 | 23.02 ± 3.44 | 0.428 |
| OS protocol (%) |  |  | 0.367 |
| GnRH agonist | 72.0% (219/304) | 68.8% (245/356) |  |
| GnRH antagonist | 28.0% (85/304) | 31.2% (111/356) |  |
| Fertilization protocol (%) |  |  | 0.875 |
| IVF | 83.9% (255/304) | 83.4% (297/356) |  |
| ICSI | 16.1% (49/304) | 16.6% (59/356) |  |

**Note:** AMH, Anti-Müllerian Hormone; FSH, follicle stimulating hormone; BMI, body mass index; OS, ovarian stimulation; GnRH, gonadotropin-releasing hormone; IVF, *in vitro* fertilization; ICSI, intracytoplasmic sperm injection.
