# Supplementary material for: Comparison of the outcomes of in vitro fertilization and embryo transfer among ethnic Chinese Yi and Han women: a multicenter retrospective cohort study
Source: PeerJ. 2026 Apr 17;14:e21145. doi: 10.7717/peerj.21145 (PMC13094553; doi:10.7717/peerj.21145)
Supplement: Supplemental Information 4 [file peerj-14-21145-s004.doc]

STROBE Statement—Checklist of items that should be included in reports of ***cohort studies***

|  | Item No | Recommendation | Page  No. | Relevant text from manuscript |
| --- | --- | --- | --- | --- |
| **Title and abstract** | 1 | (*a*) Indicate the study’s design with a commonly used term in the title or the abstract | 1 | Indicated that this has been a cohort study. |
|  |  | (*b*) Provide in the abstract an informative and balanced summary of what was done and what was found | 2 | Whether live birth rate (LBR) of *in vitro* fertilization and embryo transfer (IVF-ET) was different among Chinese Yi and Han ethnicities.  The LBR was poorer in Yi women than Han women. |
| Introduction | | | | |
| Background/rationale | 2 | Explain the scientific background and rationale for the investigation being reported | 2-3 | Previous studies have found that LBR of IVF was associated with ethnicity among American and European, while the disparities of LBR remain unknown in multiethnic Chinese. |
| Objectives | 3 | State specific objectives, including any prespecified hypotheses | 3 | Stated that the the study was to compare LBR among Chinese Yi and Han ethnicities, and the outcomes of IVF-ET may be poorer for Yi women. |
| Methods | | | | |
| Study design | 4 | Present key elements of study design early in the paper | 3 | Stated that this was a retrospective cohort study. |
| Setting | 5 | Describe the setting, locations, and relevant dates, including periods of recruitment, exposure, follow-up, and data collection | 3-6 | Described that, from January 2014 to December 2022, the patients were enrolled from three hospitals (the Reproductive Medical Centre of Sichuan Provincial Women’s and Children’s Hospital, Reproductive & Women-Children Hospital of Chengdu University of Traditional Chinese Medicine, Sichuan Jinxin Xinan Women and Children’s Hospital).  Nine years of data were reviewed.  All data came from the electronic medical record system. |
| Participants | 6 | (*a*) Give the eligibility criteria, and the sources and methods of selection of participants. Describe methods of follow-up | 3 | Stated that the eligibility criteria were Chinese Yi and Han women (determined by identity card) aged 20 ~ 45 and undergone first cycle of IVF-ET or intracytoplasmic sperm injection with non-donor oocytes and sperms.  All patients were followed up by telephone calls. |
|  |  | (*b*) For matched studies, give matching criteria and number of exposed and unexposed | 6 | Stated that propensity score matching (PSM) was performed using a 1:1 nearest-neighbor matching strategy without a caliper and ten variables were matched. |
| Variables | 7 | Clearly define all outcomes, exposures, predictors, potential confounders, and effect modifiers. Give diagnostic criteria, if applicable | 5-6 | For clinical pregnancy: the outcome was defined as the presence of gestational sac on ultrasound examination.  Live birth: defined as the delivery of a live fetus after 28^th^ gestational weeks. |
| Data sources/ measurement | 8* | For each variable of interest, give sources of data and details of methods of assessment (measurement). Describe comparability of assessment methods if there is more than one group | 5 | Live birth rate was calculated as the numbers of live birth divided by the number of embryo transfer cycles. |
| Bias | 9 | Describe any efforts to address potential sources of bias | 6 | PSM was performed to ensure that the basal characteristics of the Yi and Han women were comparable. |
| Study size | 10 | Explain how the study size was arrived at | 6 | All cases that met the inclusion criteria in the specified time period were enrolled. |
| Quantitative variables | 11 | Explain how quantitative variables were handled in the analyses. If applicable, describe which groupings were chosen and why | 6 | Continuous variables were expressed as means with standard deviation, and categorical variables were presented as numbers with percentages. |
| Statistical methods | 12 | (*a*) Describe all statistical methods, including those used to control for confounding | 6 | Continuous variables were compared by Student’s *t*-test or Mann Whitney’s U-test for normal distribution or abnormal distribution data, respectively.  Categorical variables were compared by Chi-squared or Fisher’s exact tests. |
|  |  | (*b*) Describe any methods used to examine subgroups and interactions | 6 | Stated that subgroup analyses were conducted by multiple logistic regression. |
|  |  | (*c*) Explain how missing data were addressed | 4 | Samples with missing data were excluded. |
|  |  | (*d*) If applicable, explain how loss to follow-up was addressed | 4 | Samples with missing follow-up data were excluded. |
|  |  | (*e*) Describe any sensitivity analyses | / | NA |
| Results | | | | |
| Participants | 13* | (a) Report numbers of individuals at each stage of study—eg numbers potentially eligible, examined for eligibility, confirmed eligible, included in the study, completing follow-up, and analysed | 6-7,15 | The following data have been given:  Eligibility: 51155  Included: 23313  Analysed: 23313  Figure 1. |
|  |  | (b) Give reasons for non-participation at each stage | 4-6 | The reasons for non-participation have included cancelled oocyte retrieval and cancelled fresh embryo transfer. |
|  |  | (c) Consider use of a flow diagram | 15 | As shown in Figure 1. |
| Descriptive data | 14* | (a) Give characteristics of study participants (eg demographic, clinical, social) and information on exposures and potential confounders | 6 | Basal characteristics of the patients were listed in Table 1. |
|  |  | (b) Indicate number of participants with missing data for each variable of interest | / | NA |
|  |  | (c) Summarise follow-up time (eg, average and total amount) | 3 | The average follow-up time was one year. |
| Outcome data | 15* | Report numbers of outcome events or summary measures over time | 6-7 | See Table 2, 3 and Figure 2. |
| Main results | 16 | (*a*) Give unadjusted estimates and, if applicable, confounder-adjusted estimates and their precision (eg, 95% confidence interval). Make clear which confounders were adjusted for and why they were included | 6 | An effort has been made to ensure that the basal characteristics were comparable between the two groups. |
|  |  | (*b*) Report category boundaries when continuous variables were categorized | / | NA |
|  |  | (*c*) If relevant, consider translating estimates of relative risk into absolute risk for a meaningful time period | / | NA |
| Other analyses | 17 | Report other analyses done- eg analyses of subgroups and interactions, and sensitivity analyses | 7, 15 | See Figure 2. |
| Discussion | | | | |
| Key results | 18 | Summarise key results with reference to study objectives | 7-8 | Stated that the LBR was significantly lower in Yi ethnicity compared to Han ethnicity after adjusting basal characteristic by PSM. |
| Limitations | 19 | Discuss limitations of the study, taking into account sources of potential bias or imprecision. Discuss both direction and magnitude of any potential bias | 9 | The limitations were bias in retrospective study, and lackless of some information, such as anti-Mullerian hormone and genetic information. |
| Interpretation | 20 | Give a cautious overall interpretation of results considering objectives, limitations, multiplicity of analyses, results from similar studies, and other relevant evidence | 7-9 | Stated in pages 7-9. |
| Generalisability | 21 | Discuss the generalisability (external validity) of the study results | 9 | The sample size of Yi women was small and all patients were retrospectively enrolled, and the results should be extrapolated with caution. |
| Other information | | | | |
| Funding | 22 | Give the source of funding and the role of the funders for the present study and, if applicable, for the original study on which the present article is based | 2 | This study has been jointly sponsored by the Scientific and Technological Innovation Program of Sichuan Maternal and Child Health Care Association (22FXZD01), the Scientific Research Program of Sichuan Medical Association (S22015), the Natural Science Foundation of Sichuan Province (2024NSFSC0695), the National Center for Women and Children’s Health, China CDC Maternal and Infants Nutrition and Health Research Programs (2023FYH012), and the Scientific Research Program of Chengdu Municipal Health Commission (2022155, 2023358). |

*Give information separately for exposed and unexposed groups.

**Note:** An Explanation and Elaboration article discusses each checklist item and gives methodological background and published examples of transparent reporting. The STROBE checklist is best used in conjunction with this article (freely available on the Web sites of PLoS Medicine at http://www.plosmedicine.org/, Annals of Internal Medicine at http://www.annals.org/, and Epidemiology at http://www.epidem.com/). Information on the STROBE Initiative is available at http://www.strobe-statement.org.
